# Use of endobronchial one-way valves reveals questions on etiology of spontaneous pneumothorax: report of three cases

**DOI:** 10.1186/1749-8090-4-63

**Published:** 2009-11-07

**Authors:** Wai Cho Yu, Yiu Cheong Yeung, Yiu Chang, Yuet Ling Tsang, Kwok Chu Kwong, Hau Chung Kwok, YC Gary Lee

**Affiliations:** 1 Department of Medicine & Geriatrics, Princess Margaret Hospital, Hong Kong; 2 Central Endoscopy Unit, Princess Margaret Hospital Hong Kong, Hong Kong; 3 University of Western Australia & Lung Institute of Western Australia, Sir Charles Gairdner Hospital, Perth, Australia

## Abstract

Spontaneous pneumothoraces are believed to arise when air from the supplying airway exit via a ruptured visceral pleural bleb into the pleural cavity. Endobronchial one-way valves (EBVs) allow air exit (but not entry) from individual segmental airways. Systematic deployment of EBVs was applied to three patients with secondary spontaneous pneumothoraces and persistent airleak. In all cases, balloon-catheter occlusion of the upper lobe bronchus stopped the airleak. EBVs applied to individual upper lobe segmental airways failed to terminate the airleak, which only stopped after placements of multiple EBVs to occlude all upper lobe segments. The observation questions the traditional belief of 'one-airway-one-bleb-one-leak' in spontaneous pneumothorax.

## Introduction

Secondary spontaneous pneumothoraces are common in clinical practice[1,2]. Pneumothorax is conventionally believed to result from rupture of a bleb on the visceral pleura, through which air of the supplying airway drains into the pleural cavity.

Recent data have questioned this concept. Blebs are common findings on CT of both healthy subjects and patients with lung diseases - most with no consequences. Noppen *et al *have shown that aerosolized flourescin diffusely accumulated along the visceral pleura in pneumothorax patients, and postulated that air leaks diffusely through micro-pores (pleural porosity theory) in the visceral pleura[3].

Advances on endobronchial one-way valves (EBVs) provide a unique opportunity to add insight to this debate. EBVs allow air exit (but not entry) from the segmental bronchi in which the valve is deployed. EBVs would theoretically be ideal for treating pneumothoraces, if they are results of airleak from one bleb and its supplying airway. Pooled series however showed immediate success rates of only 48%[4].

We describe the use of EBV in three patients with secondary pneumothoraces in which systematic placements of EBVs in different airway subsegments revealed findings that question the traditional 'one-airway-one-bleb-one-leak' concept.

## Patients

All three patients were Chinese males with heavy smoking history and high-resolution CT evidence of diffuse emphysematous changes (including bullae), and presented with a spontaneous pneumothorax with persistent airleak. All had very limited respiratory functions, and high operative and anesthetic risks. Non-surgical options with EBV placements were attempted. Written informed consent was obtained from the patients for publication of this report and any accompanying images.

### Case 1

**Figure 1:**
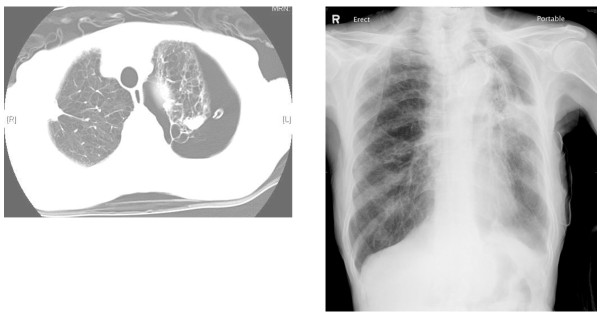
**(a, *upper*) CT scan of patient #1 prior to EBV insertion showing extensive emphysematous changes and bullae in the underlying lung, a large left pneumothorax and the inserted chest tube**. (b, *lower*) The airleak via the chest drain stopped immediately after EBV placements to occlude all upper lobe segments. The drain was removed. CXR on day 3 post-EBV insertion showed an incomplete expansion (but not total collapse) of the left upper lobe, which eventually re-expanded after bronchoscopic removal of the EBVs six weeks afterwards.

**Figure 2:**
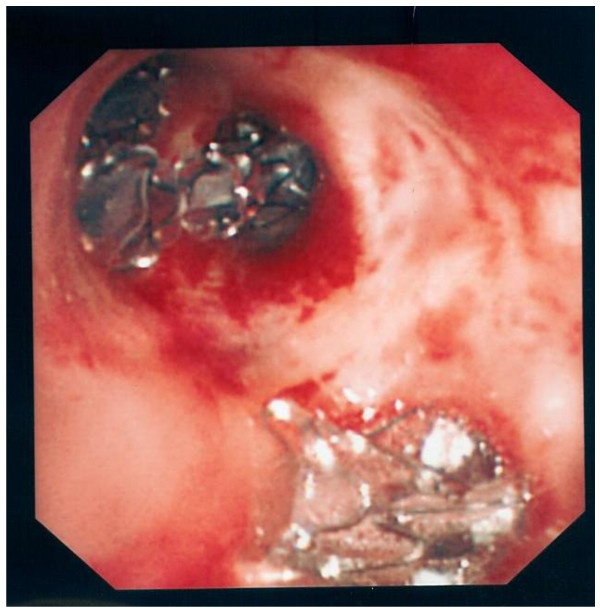
**Bronchoscopic view of the upper lobe bronchus in patient #1 showing EBVs inserted at each of the three upper lobe segments (top) and the lingular segments (bottom)**.

A 73 year-old presented with dyspnea and a near-complete left pneumothorax (fig 1a). Thoracostomy brought satisfactory lung expansion, but airleak persisted. During fibreoptic bronchoscopy (day 23), balloon-catheter occlusion of the left upper lobe (LUL) bronchus (but not individual LUL segments or left lower lobe bronchus) resulted in immediate cessation of bubbling via the chest-tube. Placement of an EBV (Emphasys, CA, USA) to occlude LB4+LB5 failed to arrest the airleak; nor did a second EBV placed in LB3, and a third in LB1. Repositioning the LB3 valve into LB2 made no impact, but the addition of a fourth EBV in LB3 (thus occluding all LUL segments) immediately stopped the airleak (fig 2). The patient was discharged two days later. Post-insertion radiograph showed incomplete re-expansion of the LUL (fig 1b) which fully resolved eventually after removal of EBVs.

### Case 2

A 61-year old with COPD and bronchiectasis had a right pneumothorax treated with bedside pleurodesis 10 years ago. He re-presented with an 80% right pneumothorax with apical adhesions which partially re-expanded with chest-tube drainage, but airleak continued. During bronchoscopy (day 34), the airleak stopped instantly with balloon-catheter occlusion of the right upper lobe (RUL) bronchus, but not the individual lobar subsegments or the bronchus intermedius. EBVs were therefore placed in RB1, RB2 and RB3. When all three devices were deployed the airleak promptly stopped - though it resumed but at a much reduced rate after 30 minutes and eventually settled completely within 72 hours. He re-presented with exacerbations of COPD twice in the subsequent six weeks but no further pneumothoraces.

### Case 3

A 70-year old man presented with chest pain and a 50% right pneumothorax which re-expanded after chest-tube insertion, but airleak persisted. At bronchoscopy (day 23), balloon-catheter occlusion of the RUL bronchus or bronchus intermedius failed to stop the airleak. Insertion of an EBV in RB1+2 and another in RB3 were technically difficult and required a second bronchoscopy the next day to achieve optimal placements. No immediate slowing of the airleak was observed, which persisted for a further 11 days before it spontaneously resolved. Follow-up CXR showed incomplete re-expansion of RUL with no pneumothorax.

None of the patients had recurrence of pneumothoraces after eight months of follow-up. All EBVs were uneventfully removed bronchoscopically six to ten weeks after insertion.

## Discussions and Conclusion

The incidence of spontaneous pneumothorax, especially from emphysema, is rising and its etiology remains controversial.

Our findings question the conventional concept that, in spontaneous pneumothorax, air leaks from a single supplying airway through a ruptured bleb on the visceral pleura. Systematic occlusion of any single segmental airway failed to terminate airleak in all three patients described. A retrospective series also showed multiple valves (mean 3.2/patient) were needed in secondary pneumothorax from COPD (n = 12 patients) and, despite that, immediate success was achieved only in 48%[4]. This helps explain the high recurrence rates (~20%) in pneumothorax patients treated with bleb resection alone[2].

Several hypotheses may explain these findings. Endobronchial lung volume reduction studies have suggested that collateral ventilation, or even alveolar-alveolar anastomoses, is underestimated[5]. Airflow through these channels increases markedly in diffuse emphysema[6]. This may explain the need of occlusion of most adjacent airways in the same lobe to arrest airleaks in patients (#1 and #2), and may also explain the failures of EBVs as occlusion of all collaterals is difficult.

Patients with pneumothorax had more significant and generalized visceral pleural abnormalities than normal subjects under autofluorescence thoracoscopy[3]. Inhaled fluorescin in these patients spread over the visceral surface rather than concentrate around one bleb[3]. Anatomical studies have raised the possibility of visceral pleural pores (of several microns wide); such porosity can account for diffuse leakage (and thus the flourescin finding and our observations).

Alternatively, there may be more than one bleb ruptured in most patients. Modern imaging techniques reveal multiple blebs as common incidental findings. It is logical that the yet unidentified trigger(s) of bleb rupture can simultaneously affect most blebs, causing leaks from multiple sites.

Establishing the etiology of secondary pneumothoraces has clinical implications: Strategies, eg pleurodesis, which treat airleak from a diffuse visceral origin are more appropriate than localized therapy (eg bleb resection). It may aid identification of suitable candidates for EBVs in future. No suitable animal models exist and future elucidation relies on collaborative clinical studies using endobronchial and imaging techniques. Whether findings on the etiology of secondary pneumothorax can be extrapolated to primary ones also requires investigation.

## Competing interests

The authors declare that they have no competing interests. Professor Lee receives a project grant from the NH&MRC Australia, and the Medical Research Council (UK).

## Authors' contributions

All authors (WCY, YCY, YC, YLT, KCK, HCK, YCGL) have made substantial contributions to conception and design, or acquisition of data, or analysis and interpretation of data. WCY and YCGL have been involved in drafting the manuscript and other authors have (YCY, YC, YLT, KCK, HCK) critically reviewed and revised it for important intellectual content. All authors have given final approval of the version to be published. All authors read and approved the final manuscript.

## Authors' information

Professor Lee is head of the Pleural Unit at the Lung Institute of Western Australia and Director of Pleural Services at Sir Charles Gairdner Hospital of Perth, Australia. He is a member of the British Thoracic Society Pleural Disease Guideline Working Group and has co-authored two books and over 80 articles on pleural diseases.
